# Death receptor 6 (DR6) is required for mouse B16 tumor angiogenesis via the NF-κB, P38 MAPK and STAT3 pathways

**DOI:** 10.1038/oncsis.2016.9

**Published:** 2016-03-07

**Authors:** X Yang, B Shi, L Li, Z Xu, Y Ge, J Shi, Y Liu, D Zheng

**Affiliations:** 1 National Laboratory of Medical Molecular Biology, Institute of Basic Medical Sciences, Chinese Academy of Medical Sciences and Peking Union Medical College, Beijing, China

## Abstract

Although death receptor 6 (DR6) is aberrantly expressed in certain cancer cell lines, its function, signaling pathway and potential clinical significance in tumor progression are not well characterized. We report here that knocking down DR6 in the mouse B16 cell line has no effect on B16 cell death *in vitro* but suppresses xenograft B16 tumor growth by preventing tumor blood vessel formation *in vivo*. Deficiency of DR6 changes cytokine expression and secretion; in particular, it inhibits the proinflammatory cytokine interleukin-6 (IL-6), which is able to induce the expression of the angiogenesis-related factors: vascular endothelial growth factor-A, platelet-derived growth factor-β, vascular endothelial growth factor-D and platelet-derived growth factor receptor-α. Further experiments demonstrate that DR6-dependent angiogenesis is involved in the IL-6/P38 MAPK and IL-6/STAT3 pathways. Our novel findings demonstrate for the first time that DR6 expression in B16 cells facilitates tumor growth by accelerating tumor angiogenesis. Moreover, these results suggest that DR6 is involved in three important intracellular pathways that lead to homeostatic angiogenesis in tumor growth.

## Introduction

Death receptor 6 (DR6, TNFRSF21) is a member of the death receptor family, which belongs to the superfamily of tumor necrosis factor receptors.^1^ As a death receptor, DR6 shows a potential anticancer role. However, Sasaroli *et al.*^2^ reported that the free form of DR6 in the serum is elevated in patients with late-stage ovarian cancer. Furthermore, high expression of DR6 has been observed in human tumors, and the overexpression of DR6 in certain tumor cells results in the elevated expression of antiapoptosis molecules.^3^ Therefore, the role of DR6 is controversial. And DR6 in tumor progression and cancer cells is rarely reported.

DR6 overexpression in HEK293 cells leads to the activation of the nuclear factor-κB (NF-κB) pathway,^3^ and NF-κB-mediated signaling is required for the maintenance of DR6 expression in Jurkat leukemia cells,^4^ which suggests that DR6 may mediate inflammatory responses via NF-κB in tumor progression.

Cytokines are mediators of communication in the inflammatory tumor angiogenesis. DR6-deficient mice show changes in cytokines production^5^ and prevent the mice brain vascular development.^6^ It has been reported that the canonical P38 MAPK and STAT3 pathway play important roles in tumor angiogenesis-related signaling,^7, 8^ and interleukine-6 (IL-6), one of the positive regulators of these pathways, is linked to the activation of tumor angiogenesis.^9, 10^ Therefore, we suggest that IL-6/P38 and IL-6/STAT3 pathways may be involved in DR6-related signaling in tumor angiogenesis.

To determine the biological function of endogenous DR6 in cancer cells and tumor progression, the B16 cancer cell line, which expresses high level of DR6, was used in our study. We generated a B16 cells implanted tumor model to track tumor growth. Further, we found that B16 tumor angiogenesis requires DR6 expression. Finally, we identified that IL-6/P38 MAPK and IL6/STAT3 pathways play critical roles in DR6-related tumor angiogenesis.

## Results

### DR6 deficiency suppresses B16 melanoma tumor growth

**Figure 1:**
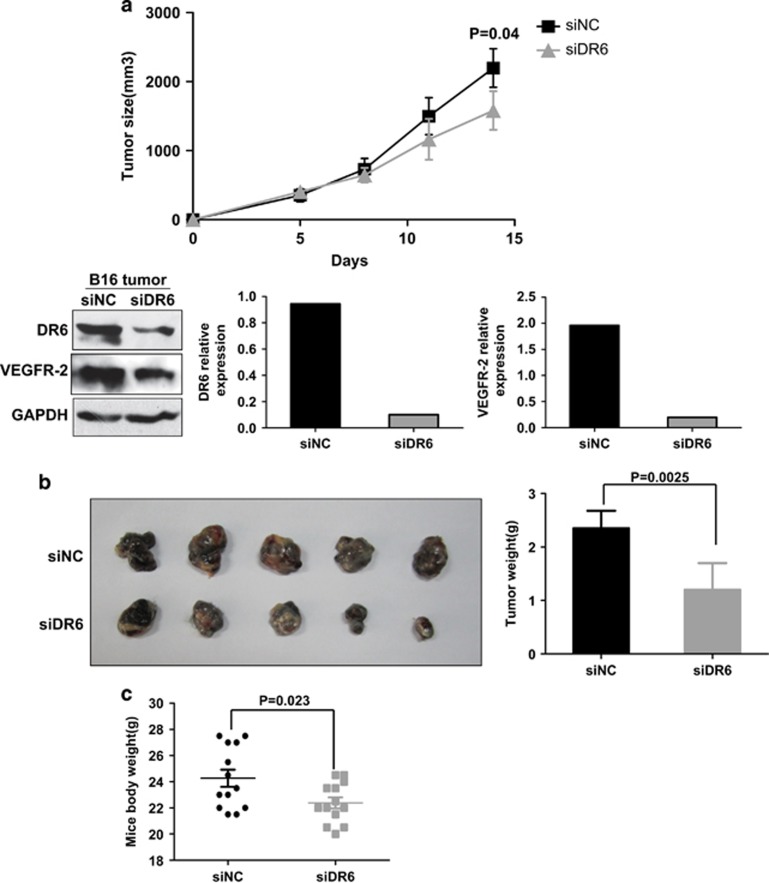
Endogenous DR6 expression in B16 cell lines is required for B16 tumor growth. (**a**) Tumor volumes. From the fifth day after implantation, tumor was measured every 3 days for a total of 14 days. Tumor size was calculated by the formula *V* (mm^3^)=length × width^2^/2 (*n*=6, *P*<0.05). Tumor cells were isolated from tumor tissues by Percoll density gradient centrifugation. The expression of DR6 and VEGF-R-2 in the B16 tumor tissue was tested by western blotting. Glyceraldehyde-3-phosphate dehydrogenase (GAPDH) was used as the loading control. (**b**) Tumor weights (*n*=6, *P*<0.05). At the end point of tumor growth measurement, the animals were killed, and the tumor tissues were excised. (**c**) Before the tumor excision, the body weight of the mice was measured (*n*=13, *P*<0.05). The error bars represent the standard deviation of the mean values obtained from triplicate experiments. *P*<0.05 is considered to be statistically significant.

DR6 expresses in mouse melanoma B16 cells, breast cancer 4T1 cells and colon cancer CT26 cells, especially highly in B16 cell lines (Supplementary Figures S1a and b). To determine the function of endogenous full-length DR6 in B16 cells, a small interfering RNA (siRNA) approach was used to knock down DR6. B16 cells were transfected with the siRNAs against DR6 (siDR6) or the negative control sequences (siNC) for 12 h, and then were implanted subcutaneously into C57BL mice. Tumor growth was measured every 3 days. As shown in Figure 1a, DR6 expression was decreased in DR6-deficient B16 tumor tissues. And knocking down DR6 (siDR6) remarkably suppressed tumor growth (Figure 1a). The expression of DR6 and VEGF-R-2 was lower in siDR6 tumors than in the control (siNC) (Figure 1a). The DR6 siRNAs reduced tumor weight (Figure 1b) as well as mouse body weight (Figure 1c) compared with the control (siNC).

### DR6 is required for tumor angiogenesis

**Figure 2:**
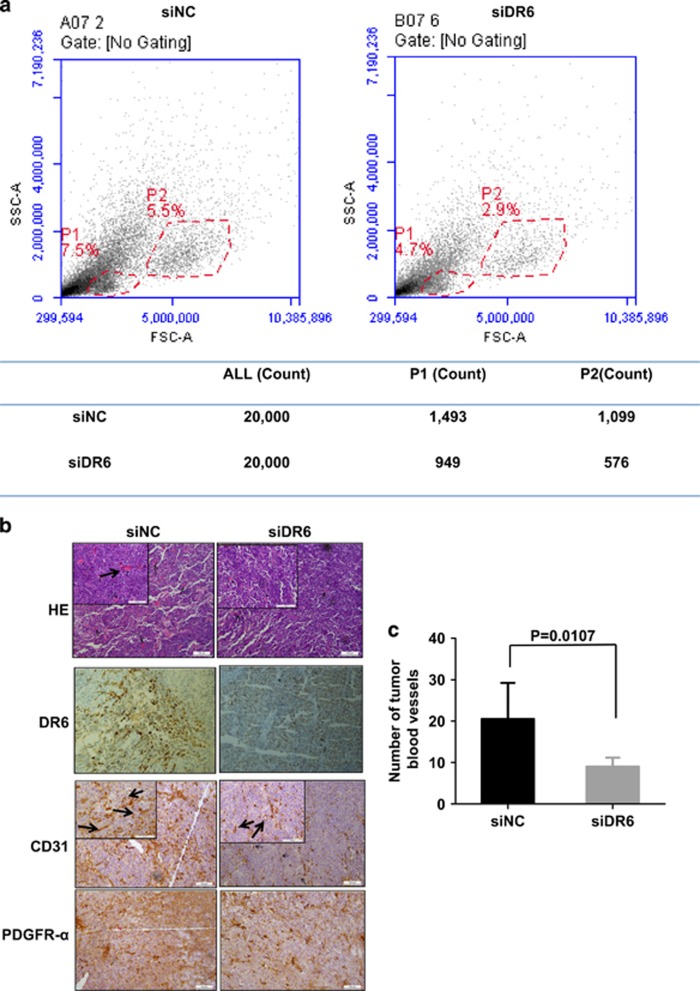
DR6 is required for tumor angiogenesis. (**a**) Immune cells were isolated by Percoll centrifugation and analyzed by flow cytometry. Label X and Y represent FSC-A and SSC-A, respectively. Gate P1 represents lymphocytes and Gate P2 represents mononuclear cells. The gated cells were counted. (**b**) Immunohistochemistry assay. The sections of DR6-deficient (siDR6) B16 tumors and controls (siNC) were stained with hematoxylin and eosin. DR6-, CD31- and PDGFR-α-specific antibodies were used to visualize blood vessels and/or as a positive control. The scale bar represents 100 μm. (**c**) Quantification of the vascular vessels. The arrows indicate representative blood vessels. The blood vessels were counted in 6/15 randomly selected fields from three mouse tumor sections under a microscope (*P*=0.01).

To test whether DR6 deficiency affects the infiltration of microenvironmental cells into melanoma tissue, immune cells were isolated from mouse tumor tissues by Percoll gradient centrifugation and analyzed by flow cytometry. As shown in Figure 2a, two populations, consisting of lymphocytes (Gate P1) and mononuclear cells (Gate P2), were observed by flow cytometry assays. Cell counting revealed that there were 50% fewer lymphocytes and mononuclear cells in the DR6 knockdown (siDR6) tumors than in the control (siNC) tumors. This suggested that a decrease of DR6 expression can prevent two subsets of immune cells infiltration into the tumor microenvironment. CD3, CTLA-4 and DC antibodies marked cells were analyzed by flow cytometry, but it showed no significant difference between siDR6 and siNC tumors (Supplementary Figure S2).

Examination of tumor tissues by hematoxylin and eosin staining and immunohistochemistry assays with an endothelial-specific antibody against CD31 indicated that there were fewer endothelial cells in the siDR6 group compared with the control group (siNC) (Figure 2b). Furthermore, the number of tumor blood vessels was decreased by approximately 50% in DR6-deficient tumor tissues compared with the control tumor tissues (Figure 2c). Additionally, there was less DR6 and platelet-derived growth factor receptor-α (PDGFR-α)-positive area in the siDR6 tumors than in the siNC tumors. To further identify whether the DR6 involves in the vascular formation, chicken chorioallantoic membrane (CAM) assays were performed. DR6 protein sequence of chicken shares highly similar conversed domain with human or mouse (Supplementary Figure S3). And DR6 antibody-treated CAM prevented vascular formation (Supplementary Figure S4) and chicken embryo size (Supplementary Figure S5). These results indicate that DR6 is required for B16 tumor angiogenesis.

### A lack of DR6 in B16 cells prevents the expression of angiogenesis-related mediators

**Figure 3:**
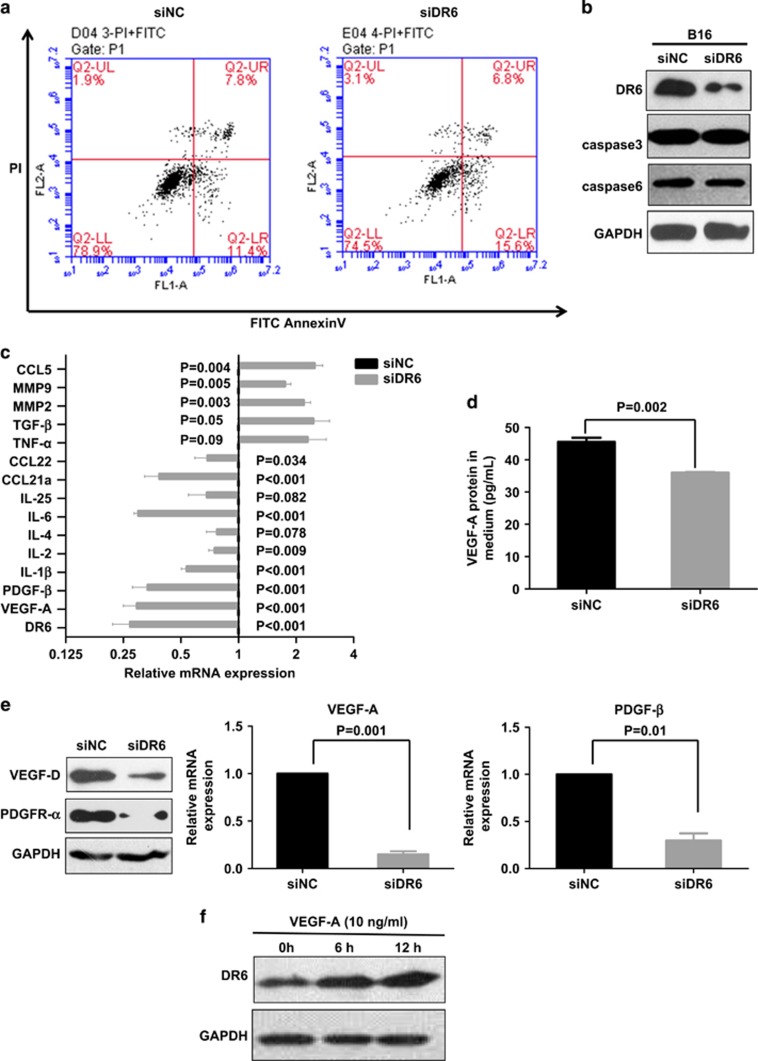
A lack of DR6 prevents the expression of angiogenesis-related mediators *in vitro*. (**a**) B16 cells were transfected with the anti-DR6 siRNA for 72 h, and then subjected to the flow cytometry assays with FITC-Annexin V/propidium iodide staining. (**b**) The expression of caspase 3 and caspase 6 was detected by western blot assays. (**c**) mRNA expression of DR6 and several cytokines in B16 cells. The mRNAs were isolated from B16 cells in siRNA-transfected for 48 h, and then examined by real-time PCR. The vertical bar in the middle of the graph is the baseline of each cytokine, which represents the siNC group. The gray bars represent the times of each mRNA expression in the siDR6-transfected group, compared with the siNC group. (**d**) VEGF-A concentration in B16 cultivated media. The cultured media were subjected to ELISA with the VEGF-A specific antibody. (**e**) Tumor cells were isolated from tumor tissues by Percoll density gradient centrifugation. The expression of VEGF-D and PDGFR-α protein and VEGF-A and PDGF-β mRNA in the B16 tumors. (**f**) Exogenous mouse VEGF-A enhances the expression of DR6 in B16 cells. Cells were treated with exogenous VEGF-A (10 ng/ml) for 0–12 h and then subjected to western blotting. GAPDH was used as the loading control. The error bars represent the standard deviation of the mean values obtained from triplicate experiments. *P*<0.05 is considered to be statistically significant.

We first tested the B16 cell apoptosis with knocking down DR6. As assessed by FITC-Annexin V and propidium iodide staining, we did not observe apoptotic cell death after knocking down DR6 expression for 72 h in B16 cells (Figure 3a). Alternative splicing of caspase 3 and caspase 6 proteins, which were reported that had been changed in DR6-expressing neuronal cells,^11, 12^ presented no change in DR6-deficient B16 cells by western blot assays (Figure 3b). These data indicate that DR6 deficiency suppresses mouse B16 tumor growth *in vivo* but has no effect on B16 cell death *in vitro*.

Cytokines are major mediators of communication between cells in the process of inflammatory tumor progression. Therefore, we examined several cytokine and chemokine mRNA expression in B16 cells. As shown in Figure 3c, IL-1β, IL-6, vascular endothelial growth factor-A (VEGF-A) and platelet-derived growth factor-β (PDGF-β) mRNA expression were dramatically decreased by knocking down DR6 expression *in vitro*. Then we next analyzed VEGF-A protein levels in the cultivated media of B16 cells by ELISA. As shown in Figure 3d, the quantity of VEGF-A decreased remarkably in the medium of siDR6-transfected B16 cells compared with that of siNC-transfected B16 cells.

The expression of VEGF-A, PDGF-β, vascular endothelial growth factor-D (VEGF-D) and PDGFR-α was determined by western blotting or real-time PCR in the DR6-deficient B16 (siDR6) tumors and the control (siNC) tumors. As shown in Figure 3e, the VEGF-D and PDGFR-α proteins and VEGF-A and PDGF-β mRNAs were significantly lower in DR6-deficient B16 tumor tissues.

DR6 expression was tested in B16 cells treated with exogenous VEGF-A (10 ng/ml). Western blot assays showed that DR6 expression was upregulated in these cells (Figure 3f), suggesting that VEGF-A enhances DR6 expression. Therefore, we conclude that VEGF-A can trigger a positive feedback to maintain homeostasis in DR6-mediated tumor angiogenesis.

### DR6 mediates tumor angiogenesis via IL-6

**Figure 4:**
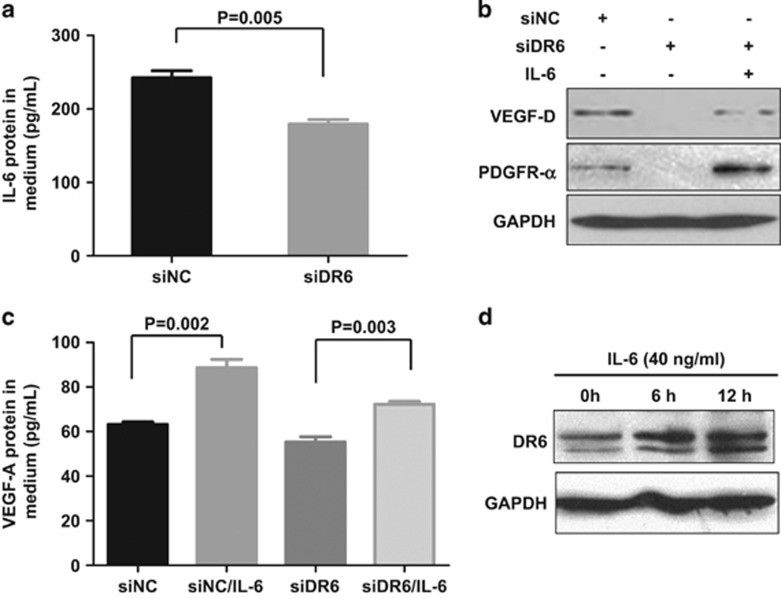
DR6 mediates tumor angiogenesis via IL-6. (**a**) Secreted IL-6 protein levels in cultivated media from B16 cells measured by ELISA. (**b**) VEGF-D and PDGFR-α protein in B16 cells were tested by rescue experiment. (**c**) VEGF-A protein in the cultured media of B16 cells was evaluated by ELISA assays. For (**b**) and (**c**), B16 cells were transfected with siDR6 or control (siNC) for 48 h and then treated with IL-6 for 12 h. The error bars represent the standard deviation of the mean values obtained from triplicate experiments. *P*<0.05 is considered to be statistically significant. (**d**) DR6 expression in B16 cells. B16 cells were treated with exogenous IL-6 (40 ng/ml) for 0–12 h and then were subjected to western blot assays. GAPDH was used as the loading control.

Knocking down DR6 of B16 cells decreased IL-6 mRNA (Figure 3c), and secreted IL-6 in B16 cells cultured media was dramatically downregulated in DR6 deficient (siDR6), compared with control (siNC) (Figure 4a), suggesting that IL-6 is involved in DR6 signaling. IL-6 signaling has been reported to play an important role in VEGF-A-dependent tumor angiogenesis.^8, 13^ Therefore, we next examined its role in DR6-mediated mouse B16 tumor angiogenesis. In B16 cells, VEGF-A, VEGF-D, PDGF-β and PDGFR-α were upregulated by IL-6 treatment on a concentration- or a time-dependent manner (Supplementary Figure S6). Not surprisingly, as shown in Figures 4b and c, the VEGF-D and PDGFR-α protein in B16 cells and the secreted VEGF-A in the cell media from DR6-deficient B16 cells were rescued by the treatment with IL-6.

To examine the feedback of IL-6 in DR6-related signaling, DR6 expression was tested in the wild-type B16 cells, which were treated with exogenous IL-6. It showed that DR6 expression was upregulated by IL-6 treatment (Figure 4d), suggesting that IL-6 is able to trigger a positive feedback loop in DR6-mediated tumor angiogenesis. These data suggest that DR6-associated B16 tumor angiogenesis is supported by IL-6.

### DR6 is involved in B16 tumor angiogenesis via the NF-κB, IL-6/P38 MAPK and IL-6/STAT3 pathways

**Figure 5:**
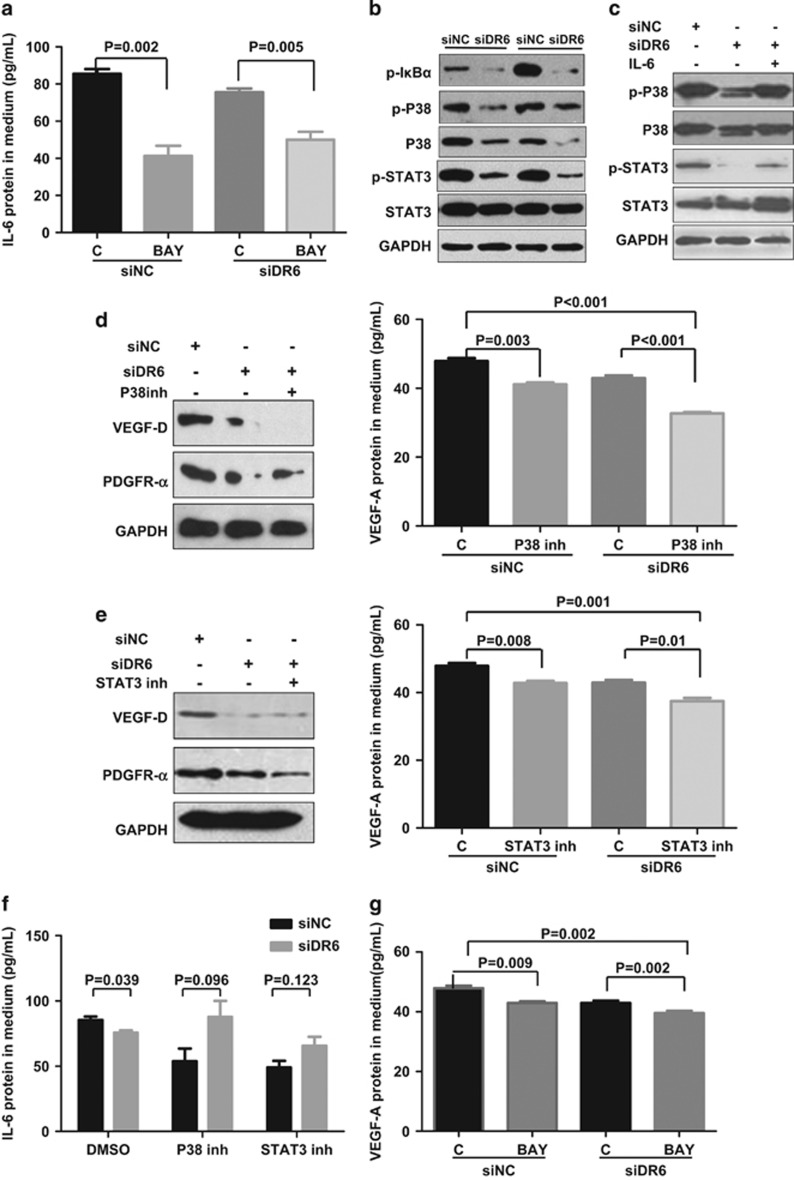
DR6 mediates tumor angiogenesis through the NF-κB, IL-6/P38 MAPK and IL-6/STAT3 pathways. (**a**) Secreted IL-6 protein in B16 cell cultured media detected by ELISA. B16 cells were transfected with siDR6 or control (siNC) for 48 h and then treated with BAY or DMSO as a solvent control (C) for 12 h. (**b**) The levels of phosphorylated IκBα (p-IκBα) phosphorylated P38 (p-P38), phosphorylated STAT3 (p-STAT3), P38 and STAT3 were evaluated by western blot assays. (**c**) The detection of p-P38, total P38, p-STAT3 and total STAT3 proteins in DR6-deficient B16 cells by rescue experiment. B16 cells were transfected with siDR6 or control (siNC) for 48 h and then treated with IL-6 for 12 h. (**d**, **e**) The VEGF-D and PDGFR-α proteins in B16 cells lysate and VEGF-A protein in B16 cell cultured media detected by western blot or ELISA assays. (**f**) After 12 h treatment with DMSO, P38 inhibitor and STAT3 inhibitor, the secreted IL-6 protein levels from B16 cells with transfected siNC and siDR6 were examined by ELISA. (**g**) The secreted VEGF-A protein in the B16 cultivated media was examined by ELISA. The error bars represent the standard deviation of the mean values obtained from triplicate experiments. *P*<0.05 is considered statistically significant.

IL-6 expression in the cultivated media from DR6-deficient B16 cells in the presence of NF-κB inhibitor BAY was largely reduced compared with control cells (Figure 5a), indicating that secreted IL-6 protein in DR6-deficient B16 cells is involved in NF-κB activation. B16 cells were treated with BAY can also prevent the expression IL-6, VEGF-A, PDGF-β mRNA and the VEGF-D and PDGFR-α protein (Supplementary Figure S7). Not surprisingly, IL-6 triggers P38 MAPK and STAT3 signaling in the wild-type B16 cells (Supplementary Figure S8).

Further experiment used exogenous IL-6 to treat CAM, the blood vessels grew after 10 h in the IL-6 treated group, compared with the control group (phosphate-buffered saline treated). And the DR6 antibodies significantly inhibited blood vessels formation (Supplementary Figure S9).

As shown in Figure 5b, p-IκBα, p-P38/P38 and p-STAT3 were dramatically inactivated in DR6-deficient B16 cells (siDR6) compared with the control cells (siNC). As shown in Figure 5c, DR6-deficient B16 cells were treated with exogenous IL-6. The result showed that IL-6 rescued the levels of phosphorylated and total proteins of P38 and STAT3, which indicated that IL-6 is the upstream regulator in DR6-related P38 and STAT3 signaling,

In the next experiment, we tested if P38 MAPK and STAT3 signaling were involved in DR6-IL6-mediated angiogenesis. Angiogenesis-related factors were measured in DR6-deficient B16 cells in the presence or absence of inhibitors of P38 or SATA3. As shown in Figures 5d and e, a P38 inhibitor (SB203580) and a STAT3 inhibitor (S3I-201) caused remarkably decrease of the VEGF-D and PDGFR-α protein expression and the VEGF-A protein secretion in DR6-deficient B16 cells. However, the secreted IL-6 in the B16 cultured media showed no significant difference with or without P38 inhibitor or STAT3 inhibitor (*P*>0.05) (Figure 5f). This result suggested that P38 MAPK and STAT3 signaling involved in DR6-IL6-mediated angiogenesis.

To determine whether NF-κB involves in DR6-associated B16 tumor vascular formation, DR6-deficient B16 cells were treated with BAY for 12 h. As shown in Figure 5g, the secreted VEGF-A protein by the BAY-treated group showed dramatically decrease in the B16 cultivated media, which indicated that the secreted VEGF-A was suppressed by NF-κB inactivation in the DR6-deficient B16 cells.

**Figure 6:**
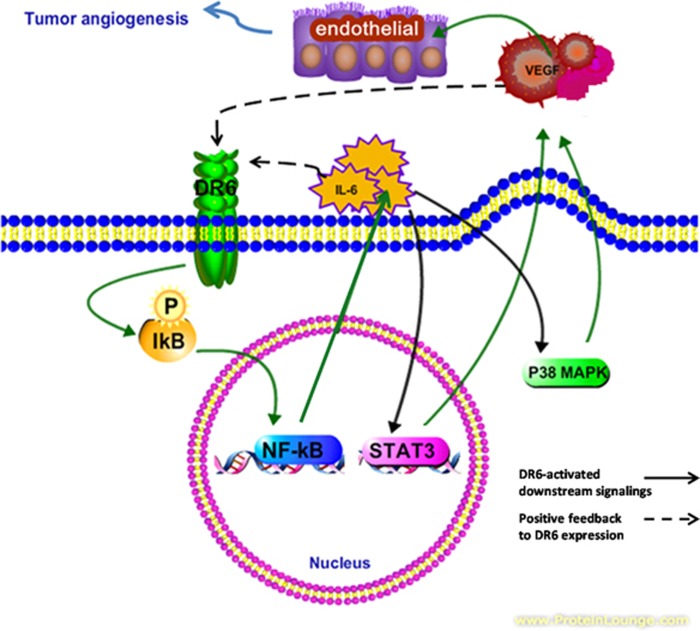
Hypothesized signaling pathway of DR6-mediated tumor angiogenesis. DR6 activates NF-κB to produce IL-6. As an important proinflammatory factor, IL-6 activates STAT3 and P38 MAPK pathways, which induce the VEGF secretion and promote the tumor angiogenesis. In return, secreted IL-6 and VEGF upregulate the DR6 expression.

Taken together, these data demonstrated that knocking down DR6 suppressed tumor angiogenesis through NF-κB, IL-6/P38 MAPK and IL-6/STAT3 signaling (Figure 6).

### The activation of IL-6 induced signaling in cancer cells is independent of IL-6R or VEGF-R expression

**Figure 7:**
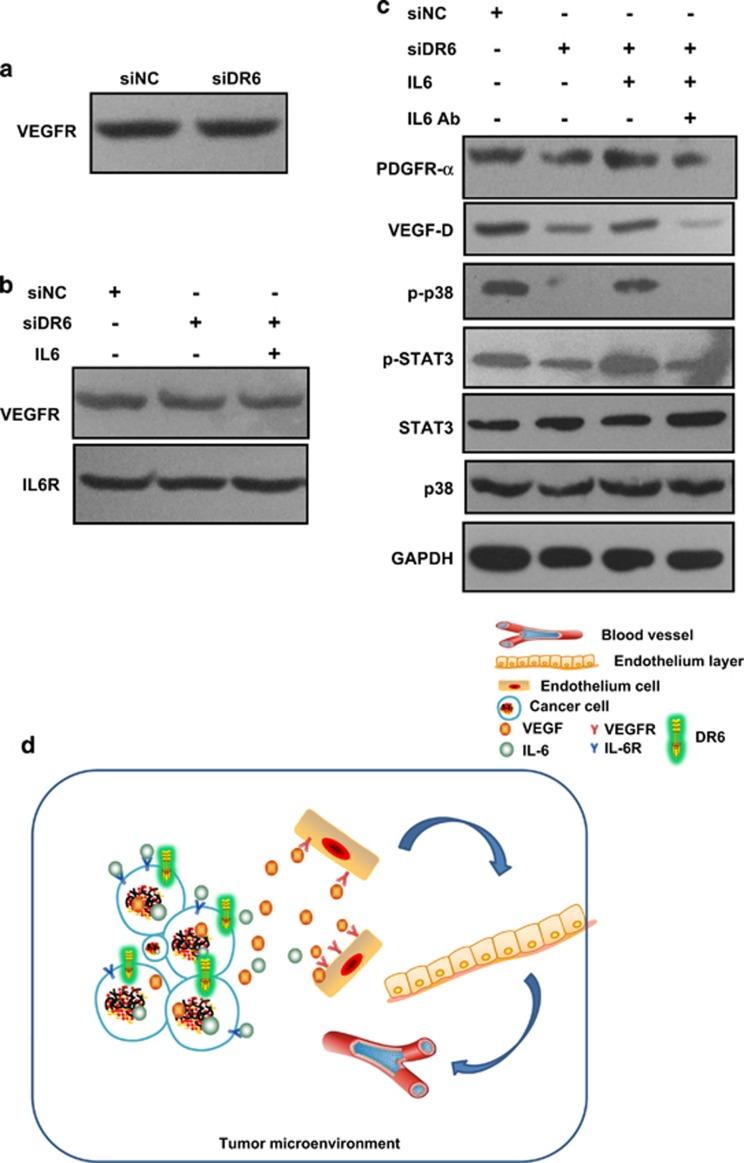
The activation of IL-6-induced signaling in cancer cells is independent of IL-6R or VEGF-R expression. (**a**) siNC- or siDR6-transfected B16 cells for 72 h, VEGF-R was detected by western blot assays. (**b**) IL-6-treated B16 cells for 6 h, VEGF-R and IL-6R were detected by western blot. (**c**) IL-6 or IL-6 antibody (Ab)-treated B16 cells for 6 h, the downstream signaling proteins were tested. GAPDH was used as the internal control. (**d**) IL-6 receptor (IL-6R) and VEGF receptor (VEGF-R) express on the tumor cell membrane. The activation of IL-6-induced signaling in cancer cells is independent of IL-6R or VEGF-R expression, but may involve in the expression of VEGF-R in endothelial cells.

No obvious change of VEGF receptor (VEGF-R) expression was detected in DR6 knockdown B16 cells (Figure 7a). Further, we used exogenous IL-6 to treat B16 cells, then analyzed IL-6 receptor (IL-6R) and VEGF-R expression. The IL-6R and VEGF-R both showed no obvious change of expression (Figure 7b). However, the downstream signaling of IL-6/IL-6R changed greatly with IL-6 or IL-6 block antibody (IL-6 Ab) treatment (Figure 7c). This result indicated that DR6 involved angiogenesis is independent of IL-6 receptor or VEGF receptor expression.

## Discussion

Based on this study, we concluded that knocking down DR6 expression inhibits mouse B16 melanoma tumor growth by suppressing the expression of the blood vessel formation-related factors VEGF-A, PDGF-β, VEGF-D and PDGFR-α, and therefore blocks tumor angiogenesis. DR6-deficient suppression of tumor angiogenesis is regulated by IL-6 via NF-κB, P38/MAPK and STAT3. IL-6 and VEGF-A upregulate DR6 expression. These results suggest that DR6 can facilitate tumor growth by accelerating tumor angiogenesis, and provide a novel model whereby DR6-dependent tumor angiogenesis is mediated by IL-6 and VEGF-A (Figure 6).

Although death receptors are typically thought to functionally interact with the cellular apoptosis machinery, different microenvironments can support or mediate distinct functions, even protect against cell death.^14^ For instance, the death receptor Fas/CD95 has been shown to promote cancer cell proliferation^15^ and to induce lung cancer growth by modifying the tumor microenvironment.^16^ Fas/CD95 can transduce both survival and death signals, depending on the strength of the stimulus.^17^ DR6 plays a regular role in promoting several tumor cell death,^1^ and recent findings clearly indicate that DR6 overexpression induces apoptosis in different cell types and that this death is dependent on mitochondrial dysfunction.^18^ In this study, the knockdown of DR6 did not affect B16 cell death but inhibited B16 tumor growth, indicating a prosurvival role of DR6 in cancer cells. And we also found DR6 overexpression played the same role with death receptor DR4 and/or DR5 (Supplementary Figure S10). However, this interesting phenomenon needs to be further clarified.

Based on previous work and our studies on DR6, we addressed the theory that DR6 plays a dual role in both survival and death during tumor progression. That means DR6 supports tumor angiogenesis, but it also can promote cancer cell to death. Studies on Fas/CD95 signal transduction in death-receptor-sensitive cells demonstrated a MAPK threshold model for cell life and death,^17^ which sheds light on the DR6-mediated cell signaling mechanism. Thus, further study requires extensive efforts to identify the ligand and components of DR6 complex in cancer cells and to determine the switching mechanism(s) between cell life and death.

At present, tumor angiogenesis is a major target of anticancer drug research. Inhibitors and antibodies that can prevent tumor growth by inhibiting tumor angiogenesis have been currently used in the clinic or tested in clinical trials. It has been reported that DR6 can serve as a serum vasculature marker in ovarian cancer development.^2^ And as a vascular biomarker, DR6 expresses highly in gynecological malignancy^19^ and breast carcinoma.^20^ DR6 knockout animals showed that DR6 is required for central nervous system-specific angiogenesis.^6^ These findings were similar to our data for DR6 in melanoma tumor angiogenesis; our model could be therefore further exploited for melanoma therapy strategies and for prognosis via vascular diagnosis.

Inflammatory mediators in the tumor microenvironment play key roles in tumor progress. Inflammatory mediators, such as interleukins and chemokines, that reside close to the tumor can affect the presence of inflammatory infiltrating in the tumor microenvironment. Therefore, the inhibition or activation of immune activities against cancer cells can be modulated by the inflammatory cells and inflammation-associated products. IL-6 leads to the initiation of Jak/STAT signaling, a pathway that turn on the chronical inflammatory cancers. The inflammatory proteins regulated by this signaling pathway may effect several tumor microenvironment-associated signalings. IL-6/STAT3 signaling in tumors often functions especially in tumor angiogenesis and influence on tumor-associated infiltrates.^21, 22^ With respect to tumor-infiltrating immune cells, elevated VEGF expression is correlated with highly enriched tumor-associated macrophage recruitment in breast cancer.^23, 24^ It has been reported that IL-6 and VEGF could be targets for several cancer therapies,^25, 26^ which pointed the crucial roles of IL-6 and VEGF in cancer research. In the present study, we demonstrated that IL-6 and VEGF were altered in a DR6-dependent manner, which pointed toward a potential correlation between DR6 and specific cell populations in the tumor microenvironment. Therefore, the relative contribution of DR6 to the changes in tumor-infiltrating lymphocytes and tumor-associated macrophages deserves further study.

Cancer behavior is likely dependent on both the driver of signaling pathways and its regulatory feedback. Tumor phenotypes seem to be less aggressive when the feedback pathway is intact. Our data supported a model whereby DR6 upregulated IL-6 and VEGF-A expression in a positive feedback manner (Figure 6), which suggested that DR6 was critical to controlling the homeostasis of tumor angiogenesis.

Usually, the activation of IL-6 or VEGF signaling is independent of the expression level of IL-6 receptor (IL-6R) or VEGF receptor (VEGF-R). Despite IL-6R or VEGF-R did not change significantly, the downstream signaling changed greatly in the normal or DR6-deficient B16 cells. Therefore, we suggested that IL-6- or VEGF-induced signaling change in cancer cells might have less influence on their receptors.

*In vivo*, the expression of VEGF-R was reduced in DR6 knockdown B16 tumor (Figure 1a). The obvious change of VEGF-R in tumors might involve in tumor microenvironment. In the mechanism of tumor angiogenesis, VEGF-R expresses on the endothelial membrane, which can bind with extracellular VEGF to promote endothelial cells growth. Therefore, in our results, we postulated that the change of VEGF-R expression occurs on the endothelial cell membrane. And the existence of DR6 in cancer cells can facilitate the endothelial cells proliferation via IL-6/VEGF-mediated mechanism during tumor angiogenesis (Figure 7d).

To sum up, DR6 plays a powerful potential role in the tumor microenvironment, especially in angiogenesis, which provides important context for the recent attempts at the therapeutic targeting of the DR6-associated signaling pathway for tumor angiogenesis and for DR6-related translational medicine.

## Materials and methods

### Cell lines and reagents

B16 mouse melanoma cells were purchased from the American Type Culture Collection (ATCC, Manassas, VA, USA) and maintained in RPMI-1640 medium (Life Technologies, Carlsbad, CA, USA) supplemented with 10% fetal bovine serum (HyClone Laboratories, Logan, UT, USA). The cells were cultured at 37 °C and 5% CO2.

The NF-κB inhibitor (BAY), P38 MAPK inhibitor (SB203580) and STAT3 inhibitor (S3I-201) were purchased from Sigma-Aldrich (St Louis, MO, USA), Beyotime (Jiangsu, China) and Merck (Darmstadt, Germany), respectively.

### DR6 knockdown experiment

Stealth siRNAs targeting DR6 were purchased from Life Technologies. The targeted DR6 mRNA sequences were as follows: (1) 5′-UAAAGGAGCCGUUUCUGCUCAGUGC-3′ (2) 5′-UUUACACUUCAUCACACUGGAAGGC-3′ (3) 5′-UACCAUUAGACUGAU ACAUUCCAGG-3′. B16 cells (1 × 10^6^) in a logarithmic growth phase were transfected with a mixture of three pairs of DR6 stealth siRNA oligos (siDR6) at a final concentration of 50 nm using Lipofectamine 2000 (Life Technologies) according to the manufacturer's instruction. The Stealth siRNA negative control duplex (siNC) (Life Technologies) was used as the negative control. Western blot assays were used to analyze the expression of DR6 protein.

### Cell death assays

Cell death was detected by the Annexin V-FITC/propidium iodide apoptosis detection kit (BD Biosciences, San Jose, CA, USA) according to the manufacturer's instructions, followed by flow cytometry (Accuri C6 Cytometer; BD Franklin Lakes, NJ, USA).

### ELISA of IL-6 and VEGF-A expression

The expression of IL-6 and VEGF-A in cell cultivated media was determined using ELISA kits (R&D Systems, Minneapolis, MN, USA) according to the manufacturer's instructions.

### Xenograft animal experiments

Six-week-old male C57BL mice were purchased from Beijing HFK Bioscience (Beijing, China). The animals were randomized into two groups (*n*=6 per group). B16 cells transfected with siDR6 or siNC were injected subcutaneously into the right dorsal flanks of the mice at 5 × 10^6^ cells per animal. The tumor sizes were measured on days 5, 8, 11 and 14 post-tumor inoculation using a caliper. Tumor volume was calculated according to the following formula: *V* (mm^3^)=length × width^2^/2. At the end point, the mice were killed using _CO2_ inhalation followed by cervical dislocation, and the tumor tissues were collected for further analysis. All the animal experiments were performed in accordance with the institutional guidelines for animal care and approved by the Institutional Animal Care and Use Committee of the Chinese Academy of Medical Sciences.

### Isolation of tumor cells and immune cells

Tumor tissues were cut into small fragments with volumes of approximately 1 mm^3^ and digested in medium RPMI-1640 with 2% fetal bovine serum, 0.05% collagenase IV (Sigma-Aldrich) and 0.005% DNase I (Sigma-Aldrich) for 1 h at 37 °C. The resulting cell suspension was passed over a 70-μm nylon filter, overlaid on 40%/70% Percoll (Pharmacia, Uppsala, Sweden) density gradient solution, and centrifuged at 400 *g*. Tumor-infiltrating immune cells then were recovered from the interphase of 40 and 70% Percoll solution, while the tumor cells remained in the upper phase of the solution.

### RNA isolation and real-time PCR

Isolated tumor cells from tumor tissue or cultured cells were lysed in TRIzol reagent (Life Technologies). Total RNAs were extracted using the method recommended by the manufacturer. Reverse transcription polymerase chain reaction was performed using the Superscript first-strand synthesis system (Life Technologies). Quantitative real-time PCR was set up with 0.5 μl of cDNA (from a total of 25 μl of each reverse transcription reaction) and primers at a concentration of 10 nm; the reactions were carried out for 40 cycles using SYBR Green and measured using an iQ5 Optical System (Bio-Rad, Hercules, CA, USA).

The primers for quantitative real-time PCR have been synthesized by Sangon Biotech (Shanghai, China). The primer sequences for mouse IL-6 are 5′-CAGAAGGAGTGGCTAAGGACCA-3′ and 5′-ACGCACTAGGTTTGCCGAGTAG-3′. The primer sequences for mouse VEGF-A are 5′-GGAGATCCTTCGAGGAGCACTT-3′ and 5′-GGCGATTTAGCAGCAGATATAAGAA-3′. The primer sequences for mouse PDGF-β are 5′-TCTCTGCTGCTACCTGCGTCTG-3′ and 5′-GGAAGTTGGCGTTGGTGCGATC-3′, and the primer sequences for mouse β-actin are 5′-TGTGCTGTCCCTGTATGCCTCT-3′ and 5′-GGAA CCGCTCGTTGCCAATAGT-3′. Quantitative real-time PCR reactions were performed in triplicate, and β-actin was used as an internal control. The cycle threshold values were converted to relative gene expression levels via the 2^−ΔΔCt^ method.

### Western blot analysis

Total proteins extracted from cells or tumor tissues were subjected to sodium dodecyl sulfate polyacrylamide gel electrophoresis, and the proteins in the gel were electrotransferred onto polyvinylidene fluoride membranes. The proteins were then probed with antibodies against DR6 (BioVision, Milpitas, CA, USA), IL-6R (Proteintech, Rosemont, IL, USA), VEGF-R-2 (Abcam, Cambridge, MA, USA), VEGF-D and PDGFR-α (Biogot, Nanjing, China), caspase 3, caspase 6, p-IκBα, p-P38, P38, p-STAT3 or STAT3 (Cell Signaling Technology, Danvers, MA, USA) and then incubated with an appropriate horse radish peroxidase (HRP)-conjugated secondary antibody (Cell Signaling Technology). The target proteins were visualized with a chemiluminescence system (Millipore, MA, USA) and exposed to X-ray film. The blot images were scanned and processed using Microsoft Office Picture Manager. Glyceraldehyde-3-phosphate dehydrogenase (GAPDH) (KangChen Biotech, Beijing, China) was used as an internal control.

### Hematoxylin and eosin staining and immunohistochemistry assay

Tumor tissues were excised, fixed in 10% neutral buffered formalin and embedded in paraffin. The paraffin-embedded tumor tissues were cut into 5 μm sections, which were then stained with hematoxylin and eosin.

For immunohistochemistry assays, the paraffin-embedded tumor sections were de-paraffinized, rehydrated and pretreated with 0.3% hydrogen peroxidase (ZSGB-Bio, Beijing, China) for 10 min. The sections were treated with Tris-EDTA buffer (pH=9) for heat-mediated antigen retrieval. After blocking with goat serum (ZSGB-Bio, Beijing, China), the sections were incubated with antibodies against CD31 (1:500; Abcam) or PDGFR-α (1:500; Biogot, Nanjing, China) for 1 h at room temperature and then incubated with an appropriate HRP-conjugated secondary antibody (ZSGB-Bio) for 30 min. Positive signals were visualized using a DAB kit (ZSGB-Bio), and the sections were counterstained with hematoxylin. Finally, the sections were examined using a Leica DMI6000B microscope, and images were captured with a Leica DFC450 C camera using Elements Software.

### CAM assays

The CAM was exposed by cutting a window (4cm^2^) on big side of a 5-day-old specific pathogen-free chicken egg. DR6 antibodies (Santa Cruz Biotechnology, TX, USA) or cytokines were placed on the CAM. The windows in the shells were sealed with adhesive tapes and the eggs incubated for 10–24 h. Representative CAMs from each group were imaged using a camera. At least three embryos were used per group.

### Statistical analysis

All the experiments repeated at least three times and all data are presented as the mean±s.e. of the mean. *P-*values were calculated via two-tailed Student's *t*-tests. *P*<0.05 was considered to be statistically significant. Statistical analyses were visually represented using GraphPad Prism (La Jolla, CA, USA).
