# Supplementary material for: Death receptor 6 (DR6) is required for mouse B16 tumor angiogenesis via the NF-κB, P38 MAPK and STAT3 pathways
Source: Oncogenesis. 2016 Mar 7;5(3):e206–. doi: 10.1038/oncsis.2016.9 (PMC4815052; doi:10.1038/oncsis.2016.9)
Supplement: Supplementary Figure Legends [file oncsis20169x2.docx]

**Supplementary fig. 1** (a). DR6 expresses in B16, 4T1 and CT26 cells. The full length (FL) and cleaved DR6 in the three of cell lines. (b). The relative comparison of quantity for FL (72kD) and cleaved (45kD) proteins in B16, 4T1 and CT26 cells were tested by Image J software.

**Supplementary fig. 2** Flow cytometry assays for CD3, CTLA-4 and DC positive cells. Isolated immune cells were subjected to the flow cytometry assays with incubated CD3, CTLA-4 and DC specific antibodies.

**Supplementary fig. 3** IL-6 up-regulates the vascular formation related in B16 cells. 20ng/mL or 40ng/mL IL-6 treated B16 cells with the time of 0h, 20h and 48h, then the B16 cell mRNA and cell lysate was collected. (a). The VEGF-A mRNA expression in IL-6 treated B16 cells. (b). The VEGF-D and PDGFR-α proteins in IL-6 treated B16 cells. GAPDH was used as the loading control. The error bars represent the standard deviation of the mean values obtained from triplicate experiments. (*** means P<0.0001, which is considered statistically significant.)

**Supplementary fig. 4** BAY inhibits the expression of IL-6 and the angiogenesis related protein. BAY or DMSO control (Con) treated B16 cells for 12 h, then the B16 cell mRNA and cell lysate was collected. (a-c). IL-6, VEGF-A and PDGF-β mRNA expression in BAY or Con treated B16 cells. (d). VEGF-D and PDGFR-α proteins in BAY or Con treated B16 cells. The error bars represent the standard deviation of the mean values obtained from triplicate experiments. (* means P<0.05, which is considered statistically significant.)

**Supplementary fig. 5** IL-6 activates P38 and STAT3. (a). 40ng/mL IL-6 treated B16 cells by a time course. The p-P38 and total P38 protein expression were tested by the western blot. (b). 40ng/mL IL-6 treated B16 cells by a time course. The p-STAT3 and total STAT3 protein expression were tested by the western blot. GAPDH was used as the loading control.

**Supplementary fig. 6** The expression of VEGF-A, VEGF-B, VEGF-D in DR6 overexpressed HEK293T cells. DR6 gene was coloned and constructed in the pCDNA3.1 vectors to overexpress DR6. pCDNA-3.1 vectors used as a negative control. TRAIL was used as a positive control. TRAIL is the ligand of DR4 and DR5, which simulate the overexpression for DR4 and DR5.

**Supplementary fig. 7** Alignment of DR6 protein sequence in *Euteleostomi*. DR6 showed highly conserved domain among *H. sapiens*, *M. musculus* and *G. gallus*. The website: *http://www.ncbi.nlm.nih.gov/homologene/?term=DR6*

**Supplementary fig. 8** The chicken chorioallantoic membrane model (CAM) assays. DR6 antibody (DR6 Ab) placed on CAM membrane, 10 hours observed the vascular formation. PBS and 0.02% BSA used as the negative control.

**Supplementary fig. 9** The observation of chicken embryos size. DR6 antibody (DR6 Ab) placed on CAM membrane, 24 hours observed chicken embryos. PBS and 0.02% BSA used as the negative control.

**Supplementary fig. 10** IL-6 involves in the DR6-related angiogenesis. PBS, DR6 Ab (concentration 1:1000), 40 ng/mL IL-6 or 40 ng/mL IL-6+ DR6 Ab treated CAM.
